# An outbreak of gastroenteritis following an international conference in Brussels, January 2015

**DOI:** 10.1186/2049-3258-73-S1-P12

**Published:** 2015-09-17

**Authors:** Cristina Valencia, Sophie Quolin, Javiera Rebolledo, Jean-Marie Tremerie

**Affiliations:** 1 Scientific Institute of Public Health, Brussels, Belgium; 2 Health Inspection Agency, Brussels, Belgium

## Background

In February 2015, organizers of an international conference in Brussels on 27-29 January reported an outbreak of gastroenteritis among its 130 attendees. We investigated the outbreak to estimate its magnitude and implement control measures.

## Methods

On 10/02/2015 we emailed a standard questionnaire to all attendees to gather demographic, clinical and exposure information and laboratory results. We defined cases as attendees with vomiting, nausea, abdominal pain or diarrhea between 27/01-02/02/2015. We interviewed food handlers and tested leftovers of the sandwiches served. Faecal specimens of five cases were tested for enteric pathogens results were reported in the questionnaire. We computed relative risks with 95% confidence intervals (CIs).

## Results

Of 107 attendees who responded, 57 were cases (AR=53%). Disease onset was between 6 hours after consuming lunch on 27 January and noon on 29 January, 54 cases occurred6 hours after the second lunch. 52 cases (91%) reported nausea, 41 (72%) abdominal pain, 38 (66%) vomiting and 27 (47%) diarrhoea. All participants attended both lunches. Participants who consumed salmon sandwiches were three times more likely to become ill (RR= 2.98 95%CI: 1.6-5.5). One stool specimen was positive for *Salmonella spp.* Leftovers of de-frosted salmon used for sandwiches during the first lunch were re-frozen then thawed for the second lunch. No food handlers reported ill. No salmon sandwich was available for testing. None of the other sandwiches tested positive for enteric pathogens.

## Conclusion

Salmon sandwiches were the probable vehicle in this large outbreak, possibly caused by Salmonella. De-freezing and re-freezing salmon leftovers may have contributed to increased pathogen concentration. Food Health authorities need to reinforce compliance with established recommendations and the education of food-handlers on proper handling and storage of leftovers.

